# The complete mitochondrial genome sequence of *Osteobrama belangeri* (Cyprinidae) and its comparison with other related Cypriniformes fish species

**DOI:** 10.1080/23802359.2019.1624206

**Published:** 2019-07-12

**Authors:** Bijay Kumar Behera, Ajaya Kumar Rout, Vishwamitra Singh Baisvar, Prasenjit Paria, Nandeibam Samarjit Singh, Sanjeev Sen Ghadei, Asim Kumar Jana, Pranaya Kumar Parida, Basanta Kumar Das

**Affiliations:** Biotechnology Laboratory, ICAR-Central Inland Fisheries Research Institute, Kolkata, India

**Keywords:** Next-generation sequencing, mitogenome, *O. belangeri*, phylogenetic tree

## Abstract

The complete mitogenome of *Osteobrama belangeri* is described using Ion Torrent (PGM sequencer), which was 16,609 bp in size comprising 13 mRNAs, teo rRNA genes, 22 tRNAs, and 926 bp as D-Loop control region, in addition to gene order and organization, being similar to most of the other related Cypriniformes fish mitogenome of NCBI databases. The all 22 tRNAs were packed into a typical clover-leaf structure. In the present study, the mitogenome has 99% similarity to the complete mitogenome sequence of *O. belangeri* mitogenome details previously and also would be helpful in understanding the phylogenetics, population genetics, and evolution of family Cyprinidae fishes.

The *Osteobrama belangeri* (Valenciennes, 1844) belongs to the order Cyrpiniformis and family Cyprinidae is a near threatened species as per IUCN status (Viswanath, 2010). It is a preferable medium sized carp in North eastern states of India. The culture of these species has been popularized in northeastern states. Apart from its delicious taste, it is having its religious importance in the state of Manipur, India for which the species has been declared as the state fish of Manipur. Apart from India, this species is also available in China and Myanmar (Barman et al. 2017), which migrates from Myanmar (Chindwin river) to the Manipur (Imphal river) (Behera et al. 2009). To plan the conservation measure and to safeguard the biodiversity, genetic characterization of fish species is vital. Whole Mitogenome information is key to biodiversity studies and similar work was carried out for other fish species (Behera, Kumari, et al. 2017; Behera, Baisvar, et al. 2017). In this back drop, whole mitogenome sequencing of *O. belangeri* was carried out. In this study, the fish sample was collected from Loktok Lake (24°30′19.82″N, 93°46′33.62″) Manipur, India in the month of July in the year 2018. This species is maintained in the fish Museum (specimen voucher no. MN-L-OB01) of ICAR-Central Inland Fisheries Research Institute, Barrackpore, Kolkata, India.

Total mitochondrial DNA was isolated from fin tissue of *O. belangeri* and sequenced using 2nd generation Ion Torent PGM Sequencing platform (Life technologies, Waltham, MA). A total of 123,417 reads were obtained and compared with 19 number of fish mitogenome. Mapped reads were *de novo*-assembled by Torrent Mapping Alignment Program (TMAP) using Torrent Suite software version 4.0 (Ion Torrent, Life Technologies, La Jolla, CA). The minimum evolutionary Phylogenetic tree was constructed using method (Rzhetsky and Nei 1992).

The complete Mitogenome of *O. belangeri* is 16,609 bp having GenBank Accession No. MK749691 with 13 protein-coding genes, two rRNA genes, 22 tRNA genes, and a 926-bp-long control region. The major number of genes was encoded on the H-strand except tRNA^Glu^, tRNA^Gln^, tRNA^Ala^, tRNA^Tyr^, tRNA^Pro^, tRNA^Asn^, tRNA^Cys^, tRNA^Ser^, and ND6 which were encoded on L strand and all 22 tRNAs were packed into a typical clover-leaf structure. In general, the base composition of *O. belangeri* mitogenome was A (33.10%), T (27.60%), C (24.40%), and G (15.00%) and was A + T (60.7%) rich similar to the other related mitogenomes. The evolutionary history of *O. belangeri* was established with closely related 19 species of Cyprinadae using the Minimum Evolution method (Rzhetsky and Nei 1992) and the optimize tree with a sum of total branch length (0.72897974) and the replicate trees in percentage, where associated Taxa clusters with branch length present above the branches. The Minimum evolutionary Phylogenetic tree was drawn using MEGA 6.0 (McAllister Ave, USA) (Tamura et al. 2013) using 19 related Cypriniformes fish species mitogenome from NCBI database. The *O. belangeri* is very close to the cluster of *Systomussarana sarana*, *Systomus orphoides*, *Barbus eburnunsis*, and *Enteromius guirali* than other related Cypriniformes fish species (Figure 1).

**Figure 1.**
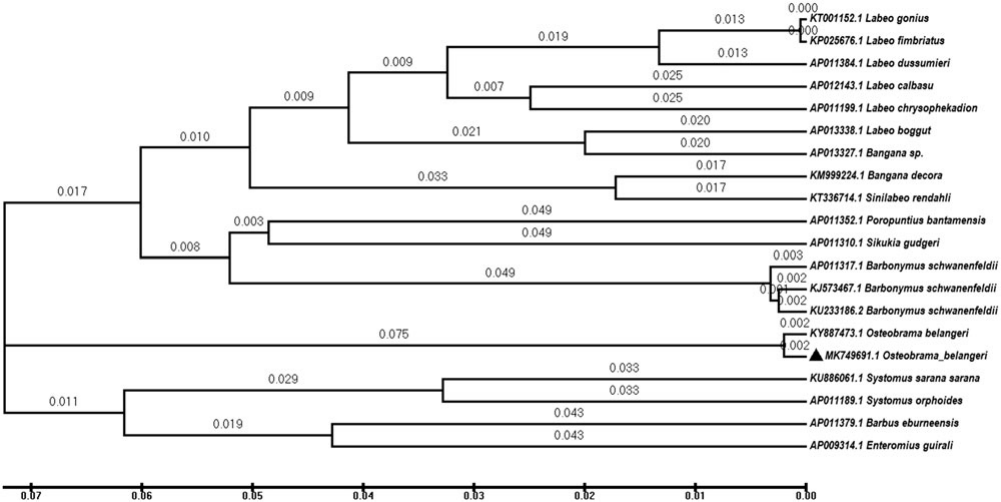
Minimum evolutionary Phylogenetic tree of Cypriniformes by taking 19 related fish mitogenome sequences (all parameters were used as default with gap opening penalty 15, gap extension penalty 6.66, and multiple alignment parameters set as gap opening penalty of 15, gap extension 6.66, DNA weight matrix IUB, and transition weight 0.5 for alignment.

## References

[CIT0001] Barman AS, Singh M, Pandey PK 2017 Complete mitochondrial genome of near threatened fish species *Osteobrama belangeri* (Cypriniformes: Cyprinidae). Mitochondrial DNA B. 2:300–301. 10.1080/23802359.2017.1331327 PMC7800496 33473805

[CIT0002] Behera BK, Kumari K, Baisvar VS, Rout AK, Pakrashi S, Paria P, Jena JK 2017 Complete mitochondrial genome sequence of Indian medium carp, *Labeo gonius* (Hamilton, 1822) and its comparison with other related carp species. Mitochondrial DNA A DNA MappSeq Anal. 28:7–8. 26710185 10.3109/19401736.2015.1106517

[CIT0003] Behera BK, Baisvar VS, Kumari K, Rout AK, Pakrashi S, Paria P, Rao AR, Rai A 2017 The complete mitochondrial genome of the *Anabas testudineus* (Perciformes, Anabantidae) and its comparison with other related fish species. Mitochondrial DNA A DNA MappSeq. 28:161–162. 10.3109/19401736.2015.1115490 26709978

[CIT0004] Behera BK, Das P, Ngachan SV 2009 Strategies for improving fish production in Loktak Lake. Ecology, Aquatic Bio-resources and Conservation of Wetlands of North East India. Akansha Publishing House, New Delhi; p. 29–40.

[CIT0005] Rzhetsky A, Nei M 1992 A simple method for estimating and testing minimum evolution trees. Mol Biol Evol. 9:945–967.

[CIT0006] Tamura K, Stecher G, Peterson D, Filipski A, Kumar S 2013 MEGA6: Molecular evolutionary genetics analysis version 6.0. Mol Biol Evol. 30:2725–2729. 24132122 10.1093/molbev/mst197 PMC3840312

[CIT0007] Vishwanath W 2010 *Osteobrama belangeri* The IUCN Red List of Threatened Species. 2010:e.T168218A6467894. 10.2305/IUCN.UK.2010-4.RLTS.T168218A6467894.en

